# Plasma vesicular miR-155 as a biomarker of immune activation in antiretroviral treated people living with HIV

**DOI:** 10.3389/fimmu.2022.916599

**Published:** 2022-08-29

**Authors:** Wilfried Wenceslas Bazié, Julien Boucher, Benjamin Goyer, Isidore Tiandiogo Traoré, Dramane Kania, Diane Yirgnur Somé, Michel Alary, Caroline Gilbert

**Affiliations:** ^1^ Axe de Recherche Maladies Infectieuses et Immunitaires, Centre de Recherche du CHU de Québec-Université Laval, Québec, QC, Canada; ^2^ Programme de Recherche sur les Maladies Infectieuses, Centre Muraz, Institut National de Santé Publique, Bobo-Dioulasso, Burkina Faso; ^3^ Institut Supérieur des Sciences de la Santé, Université Nazi Boni, Bobo-Dioulasso, Burkina Faso; ^4^ Axe de Recherche Santé des Populations et Pratiques Optimales en Santé, Centre de Recherche du CHU de Québec-Université Laval, Québec, QC, Canada; ^5^ Département de Médecine Sociale et Préventive, Faculté de Médecine, Université Laval, Québec, QC, Canada; ^6^ Institut National de Santé Publique du Québec, Québec, QC, Canada; ^7^ Département de Microbiologie-Infectiologie et d’Immunologie, Faculté de Médecine, Université Laval, Québec, QC, Canada

**Keywords:** biomarker, extracellular vesicles, HIV-1, immune activation, MicroRNA, miR-29a, miR-146a, miR-155

## Abstract

People living with HIV (PLWH), despite suppression of viral replication with antiretroviral therapy (ART), have high morbidity and mortality due to immune activation and chronic inflammation. Discovering new biomarkers of immune activation status under ART will be pertinent to improve PLWH quality of life when the majority will be treated. We stipulate that plasma large and small extracellular vesicle (EVs) and their microRNA content could be easily measured biomarkers to monitor immune activation in PLWH. Venous blood samples from n = 128 ART-treated PLWH with suppressed viral load (≤ 20 copies/mL) and n = 60 HIV-uninfected participants were collected at five testing or treatment centers of PLWH in Burkina Faso. Large and small plasma EVs were purified, counted, and the mature miRNAs miR-29a, miR-146a, and miR-155 were quantified by RT-qPCR. Diagnostic performances of large and small EVs miRNAs level were evaluated by receiver operating characteristic (ROC) curve analysis and principal component analysis (PCA). Among the EVs microRNA measured, only large EVs miR-155 copies distinguished PLWH with immune activation, with AUC of 0.75 for CD4/CD8 < 1 (95% CI: 0.58–0.91, P = 0.0212), and 0.77 for CD8 T cells ≥ 500/µL (95% CI: 0.63–0.92, P = 0.0096). In addition, PCA results suggest that large EVs miR-155 copies may be a biomarker of immune activation. Since miR-155 may influence immune cell function, its enrichment in large EV subpopulations could be a functional biomarker of immune activation in PLWH on ART. This measure could help to monitor and diagnose the immune activation with more accuracy.

## Introduction

In persons living with the human immunodeficiency virus (HIV), a condition characterized by generalized immune activation and chronic inflammation often develops even after successful treatment with antiretroviral drugs (1). This systemic immunopathogenesis is self-sustained and persists as a residual long-term sequela even though the plasma viral titer has been reduced to undetectable levels (1, 2). This chronic condition is associated with a high risk of developing non-AIDS morbidity and multimorbidity, leading to lower life expectancy (3, 4). More and more medical practitioners recognize that viral suppression alone does not ensure the long-term wellbeing of people living with HIV (PLWH) (5). The scientific community is searching for new biomarkers that will make this practical in the clinical setting. The task is daunting, given the complexity of molecular and cellular parameters to be investigated (1).

In recent years, the isolation and analysis of tumor-derived biomarkers such as extracellular vesicles (EVs) in body fluids, now known as liquid biopsy (6), is becoming an increasingly important tool for oncological diagnostics and prognostics. These tumor-derived materials represent a source of genomic, proteomic, and lipidomic information that are helpful for early diagnosis, risk stratification, disease monitoring, and selection of personalized treatment for cancer patients (7–9). Adopting this new category of biomarkers in infectious diseases could enhance our understanding of the pathophysiology of agents such as HIV.

EVs contain proteins and microRNA that can regulate gene expression or physiological responses in recipient cells (10). Previous studies have shown that circulating EVs in HIV-positive individuals are more abundant than healthy controls (11, 12). MicroRNA molecules such as miR-29a, miR-146a, and miR-155, in plasma or cell-bound, have been associated with HIV replication and latency (13, 14), inflammation and immune activation (15, 16).

Few studies have explored plasma EV miRNA content during HIV infection, and little is known about its possible relationship to the chronic immune activation and inflammation state seen in patients on antiretroviral therapy (ART). We have shown previously that plasma EV subtypes differ in their contents of miR-29 a and b, miR-92, miR-155 and miR-223 and that HIV patients can be distinguished from uninfected control subjects based on EV miRNA content (17).

In view of the damaging effects of HIV-associated persistent immune activation, identifying the regulators involved is urgent. Based on our previous observations and on published data, we focus here on microRNA involved in the regulation of immune cell functions and processes such as immune activation and inflammation. MicroRNA 155 has been described as unique in its ability to shape the transcriptome of activated myeloid and lymphoid cells that control diverse biological functions ranging from inflammation to immunological memory (18–22). It is required for maintaining lymphocyte homeostasis and normal immune function (18, 22, 23) and could alone explain all the immune disorders observed during HIV infection. MicroRNA 146a is believed to down-regulate inflammatory immune responses by targeting tumor necrosis factor receptor-associated factor 6 (TRAF6) and interleukin (IL)-1 receptor-associated kinase 1 (IRAK-1) (24). Together, 146a and 155 are among the most studied miRNA for their critical role in the regulation of lymphocyte development, differentiation, and function as well as in the control of the innate and adaptive immune processes and deregulations thereof (24–26). MicroRNA 29a is used as control because it is associated more with HIV replication than with immune activation. In PBMC, its level is inversely correlated with viremia and its overexpression is associated with inhibited viral replication and could promote viral latency (13, 14).

In this study, we investigated the expression levels of miR-29a, miR-146a and miR-155 in plasma EVs and evaluated them as quantitative markers of immune activation in PLWH. Since EV function is correlated somewhat with its size category, we followed MISEV 2018 guidelines (27) and used a purification method that separates large vesicles from smaller ones (11, 17).

## Materials and methods

This section has already been published previously (28).

### Study subjects

HIV-infected participants were recruited by referral from five HIV testing or treatment centers in Bobo Dioulasso and Ouagadougou (Burkina Faso). These included the two “Yerelon” clinics of the “Centre Muraz” specializing in female sex workers follow-up, the “Association African Solidarité (AAS)” center serving a male clientele that engages in homosexual activity, as well as two day hospitals, CHU Souro Sanou (Bobo-Dioulasso) and CHU Yalgado Ouédraogo (Ouagadougou), serving a general HIV clientele. The HIV-negative participants were recruited in the Yerelon clinics, which also offer HIV testing services. All subjects were anonymous volunteers and provided written informed consent to participate in the study.

### Quantitation of HIV-1 RNA, CD4 and CD8 T lymphocytes

The HIV-1 viral load was measured using the COBAS^®^ AmpliPrep/COBAS^®^ TaqMan^®^ Real-Time PCR assay (TaqMan, Roche Diagnostics, Mannheim, Germany), which targets two highly conserved regions of the HIV-1 genome and has a detection limit of 20 copies/mL. Absolute counts of CD4+ T and CD8+ T lymphocytes were obtained using a BD FACSCount™ System flow cytometer (Becton Dickinson, San Jose, CA, USA).

### Purification of extracellular vesicles

Purification of EVs was performed as described previously (11, 17). Briefly, blood obtained by venipuncture with citrate as an anticoagulant was centrifuged for 10 min at 400× *g* at room temperature. The plasma was centrifuged for 10 min at 3000× *g* to obtain platelet-free-plasma and stored at −80°C until analysis. Thawed platelet-free plasma (250 µL) was treated with proteinase K (1.25 mg/mL, Ambion™, Thermo Fisher Scientific, Waltham, MA, USA) for 10 min at 37°C. This pretreatment of plasma with proteinase K is described to critically reduce the amount of non-EV proteins (albumin and the apolipoproteins A-1 and B), which can be co-purified with EV. This digestion step destroys proteins and their cargo (mRNA and miRNA), and they were removed with the washing step. Large EVs were purified by centrifuging proteinase-K-pretreated plasma for 30 min at 17,000× *g* at room temperature. The supernatant was mixed with 63 µL of ExoQuick-TC™ (SBI *via* Cedarlane, Burlington, ON, Canada) in an Eppendorf tube and maintained at 4°C overnight. The large EV pellet was resuspended in 250 µL of microfiltered (0.22-µm pore size membrane) 1x phosphate-buffered saline (WISENT Bioproducts, Saint-Jean-Baptiste, QC, Canada) and centrifuged for 30 min at 17,000× *g*. The supernatant was discarded, and the washed large EV pellet was resuspended in 250 µL of PBS and kept at 4°C. Small EVs were obtained from the ExoQuick-TC™ precipitation after centrifuging for 30 min at 1500× *g*. The ExoQuick precipitate was centrifuged for 30 min at 1500× *g*. The supernatant was discarded, and the pellet was washed in PBS and centrifugated for 5 min at 1500× *g* to obtain small EVs, which were resuspended in 250 µL of PBS by vortex mixing and kept at 4°C. The size of the EV was determined by hydrodynamic radius measurements using a Zetasizer Nano S (Malvern Instruments, Ltd., Malvern, United Kingdom). This technique is based on the light scattering intensity due to the Brownian motion of EVs, characterized by a diffusion constant (29). The size distribution is obtained from the distribution of diffusion constants using the Einstein–Stokes equation (29). The measurements were made at a fixed position with an automatic attenuator and at a controlled temperature. One hundred microliters of EV suspension were used for each sample, and two measurements were averaged. We have submitted all relevant data of our experiments to the EV-TRACK knowledgebase (EV-TRACK ID: EV220124) (30).

### EV flow cytometry analysis

Purified EVs were stained with the lipophilic fluorescent carbocyanine dye DiD (DiIC18(5) solid: 1,1′-dioctadecyl-3,3,3′,3′-tetramethylindodicarbocyanine 4-chlorobenzenesulfonate salt (Invitrogen™, Carlsbad, CA, USA), and the vesicular or cell-permeable dye CFSE (carboxyfluorescein diacetate succinimidyl ester, Invitrogen™, Carlsbad, CA, USA). DiD and CFSE were prediluted, respectively, 1/100 and 1/500 with filtered (0.22 µm) PBS 1X + EDTA (100 µM for the final concentration). Forty microliters of DiD diluted solution (final dilution 1/200, 1 µg/mL) was added to 10 µL of EV suspension. After 5 min at 37°C, 50 µL of CFSE (diluted 1/1000, 1 µg/mL) were added. After 15 min (37°C), the staining was fixed by adding 0.02% Pluronic F-127 (Invitrogen™, Carlsbad, CA, USA) solution. Next, 100 µL of 4% paraformaldehyde (Fisher scientific™, Ottawa, CA, USA) solution was added and incubated for 20 min, and the tube was completed to 400 µL with 200 µL of filtered PBS 1X. Then, 5 µL of 15-µm count beads (Polybead^®^ Microspheres 15 µm, Polysciences, Inc., Warrington, PA, USA) were mixed in by vortex. A flow cytometry method described previously (11, 17) was used to count EVs in a FACS Canto II Special Order Research Product cytofluorometer equipped with forward scatter coupled to a photomultiplier tube (FSC-PMT) with the “small particles option” (BD Biosciences, Franklin Lakes, NJ, USA). Gating strategies for EV identification and analysis are described elsewhere (17).

### MicroRNA quantification

EV suspension (100 µL) was diluted 3:1 in TRIzol LS (Ambion™, Life Technologies, Carlsbad, CA, USA) and maintained at −80°C. Total RNA was extracted, mixed with 10 µL of diethylpyrocarbonate water, and quantified in 1 µL using a BioDrop-μLITE kit (Isogen Life Science, Utrecht, The Netherlands). After treatment with RNase-free DNase I (Ambion™ Life Technologies), 100 ng of RNA was reverse transcribed using a HiFlex miScript RT II kit according to the manufacturer’s instructions (Qiagen, Hilden, Germany). Mature miR-29a (#MS00003262), miR-146a-5p (#MS00006566), and miR-155-5p (#MS00031486) were detected by quantitative polymerase chain reaction (RT-qPCR) using an miScript primer assay kit and miScript SYBR Green PCR kit (Qiagen). Mature microRNA as cDNA was amplified in a CFX384 Touch Real-Time PCR Detection System (Bio-Rad, Hercules, CA, USA) using 40 cycles of 94°C for 15 s, 55°C for 30 s, and 72°C for 30 s. Reaction specificity was confirmed using the melt curve procedure (65–95°C, 0.5°C per 5 s) at the end of the amplification protocol according to the manufacturer’s instructions. A microRNA control (miRTC control # MS00000001) was used in conjunction with the miScript II RT Kit to ensure the quality of the reverse transcription during the qPCR step. A standard curve was used for the absolute quantification of microRNA. Quantifying miRNA was expressed as copies per µg of RNA and copies per vesicle, as described previously (11).

### Statistical analysis

All analyses were performed with GraphPad Prism 8 (GraphPad Inc, San Diego, CA, USA) and RStudio Version 1.4.1103 (Integrated Development for R. RStudio, PBC, Boston, MA, USA, 2020, http://www.rstudio.com/) to build correlation matrices and principal component analyses. Participant demographic and clinical characteristics were presented as a proportion or median with an interquartile range (IQR) and tabulated (**Table 1**). Initial tests of normality and log normality indicated that the EV count and miRNA content expressed as copies/µg and copies/vesicle both fit a lognormal distribution. All values were therefore transformed to logarithms. Data were then analyzed assuming a Gaussian distribution using parametric tests, and ordinary one-way ANOVA corrected for multiple comparisons using the Tukey test for three or more group comparisons. In all graphs, the results are presented as the geometric mean with geometric standard deviation factor. Pearson parametric correlation tests were performed to build correlation matrices in R. The diagnostic value of the EV miRNA content was evaluated using receiver operating characteristic (ROC) curves. Analyses were performed using the Wilson/Brown method, and the results were tabulated. In **Table 1**, the chi-square test if applicable or Fisher’s test was used to compare categories (fixed effect variables) and a t-test was used for continuous variables. A *p*-value less than 0.05 was considered statistically significant.

**Table 1:** Demographic and Clinical Characteristics of study participants.

Control (*n*=60)		*HIV+ ART-treated for more than six months with undetectable VL (*n*=128)*	P value	HIV+ ART-treated for more than six months with undetectable VL (n = 128)								
				CD4 ≥ 500 (*n* = 66)	CD4 < 500 *(n* =62)	P value	Ratio CD4/CD8 ≥ 1 (*n* = 41)	Ratio CD4/CD8 < 1 (*n* = 87)	P value	CD8 ≥ 500 (*n* = 102)	CD8 < 500 (*n* = 26)	P value
Male: n (%)	14 (23.3)	31 (24.2)	0.8945	12 (18.2)	19 (30.7)	0.1000	10 (24.4)	21 (24.1)	0.9752	21 (20.6)	10 (38.7)	0.0576
Female sex workers: n (%)	.	63 (49.2)	.	34 (51.5)	29 (46.8)	0.7809	22 (53.7)	41 (47.1)	0.1514	50 (49.0)	13 (50.0)	0.0038
Men who have sex with men: n (%)	.	20 (15.6)	.	9 (13.6)	11 (17.7)		9 (21.9)	11 (12.6)		11 (10.8)	9 (34.6)	
Age (years): median (IQR)	27 (22–32)	38 (32–45)	< 0.0001	36 (30–45)	39 (33–45)	0.5236	36 (31–42)	39 (32–45)	0.3857	39 (31–45)	34 (28–41)	0.0188
< 20	3 (5.0)	2 (1.7)	< 0.0001	1 (1.5)	1 (1.6)	0.8623	.	2 (2.3)	0.6887	1 (1.0)	1 (3.8)	0.1440
20 -29	34 (56.7)	16 (12.5)		10 (15.15)	06 (9.7)		7 (17.1)	9 (10.3)		10 (9.8)	6 (23.1)	
30 -39	19 (31.7)	49 (38.3)		25 (37.9)	24 (38.7)		16 (39.0)	33 (37.9)		38 (37.2)	11 (42.3)	
40 - 49	4 (6.7)	47 (36.7)		22 (33.3)	25 (40.3)		15 (36.6)	32 (36.8)		40 (39.2)	7 (26.9)	
≥ 50	.	14 (10.9)		8 (12.1)	6 (9.7)		3 (07.3)	11 (12.6)		13 (12.8)	1 (3.8)	
HIV duration (months): median (IQR)	NA	55	.	87	50	0.0629	48	58	0.5508	59	39	0.5338
	NA	(24–120)		(24–139)	(23–108)		(30–120)	(24–120)		(22–120)	(22–123)	
CD4 T cell count (Cells/µL): median (IQR)	992	513	< 0.0001	750	368	< 0.0001	757	429	< 0.0001	542	467	0.0371
	(794–1276)	(386–755)		(602–937)	(309–428)		(608–939)	343–581)		(404–809)	(351–639)	
CD8 T cell count (Cells/µL): median (IQR)	584	736	0.0003	826	615	0.0234	575	822	< 0.0001	819	430	< 0.0001
	(447–715)	(536–1093)		(620–1180)	(502–845)		(430–732)	(588–1213)		(631–1190)	(322–471)	
Ratio CD4/CD8: median (IQR)	1.8 (1.4–2.1)	0.7 (0.5–1.0)	< 0.0001	1.0 (0.7–1.3)	0.5 (0.3–0.8)	< 0.0001	1.3 (1.1–1.6)	0.6 (0.4–0.8)	< 0.0001	0.6 (0.4–0.9)	1.2 (0.8–1.6)	< 0.0001
ART duration (months): median (IQR)	NA	38 (21–96)	.	41 (24–114)	33 (16–79)	0.0552	36 (22–88)	41 (20–97)	0.9608	40 (23 – 96)	36 (19 – 95)	0.8186

ART, antiretroviral therapy; CD4, CD4 T cell count; CD8, CD8 T cell count; HIV, Human Immunodeficiency Virus; IQR, inter quartile range; VL, viral load.

## Results

### MiR-155 in large EVs is diagnostic marker of HIV-induced immune activation

**Figure 1:**
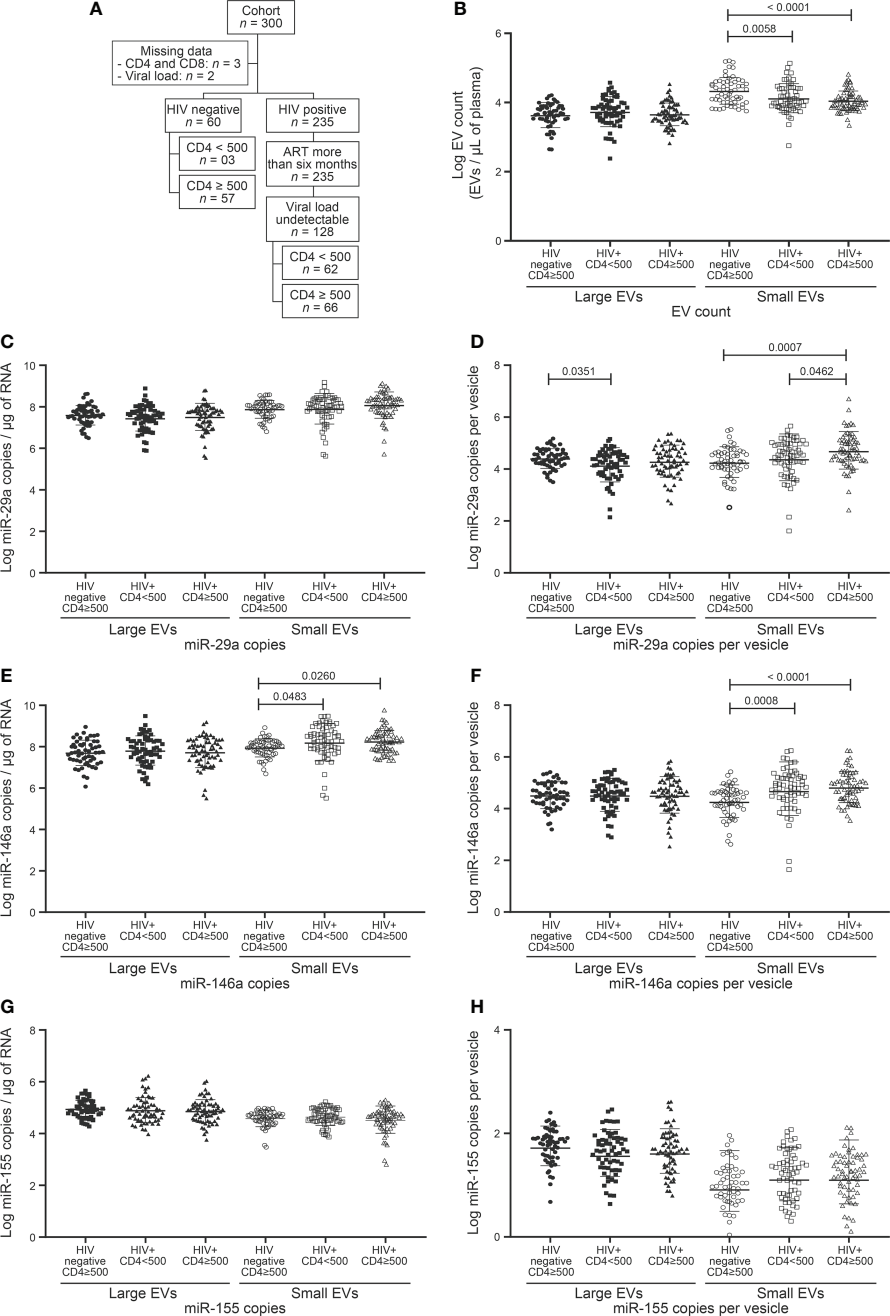
EVs count and miRNAs expression in HIV negative and HIV infected with undetectable viral load study participants follow to CD4 T cells count. **(A)** Flow chart of study participants included in the analysis following CD4 T cells count. **(B)** Large and small EVs particles number quantification in flow cytometry analysis. **(C, D)**, **(E, F)**, and **(G, H)** present respectively EVs miR-29a, miR-146a, and miR-155 quantification and expression as copies per µg of RNA and copies per vesicle. An ordinary one-way ANOVA with Tukey’s multiple comparisons test was used for comparison between groups.

**Figure 2:**
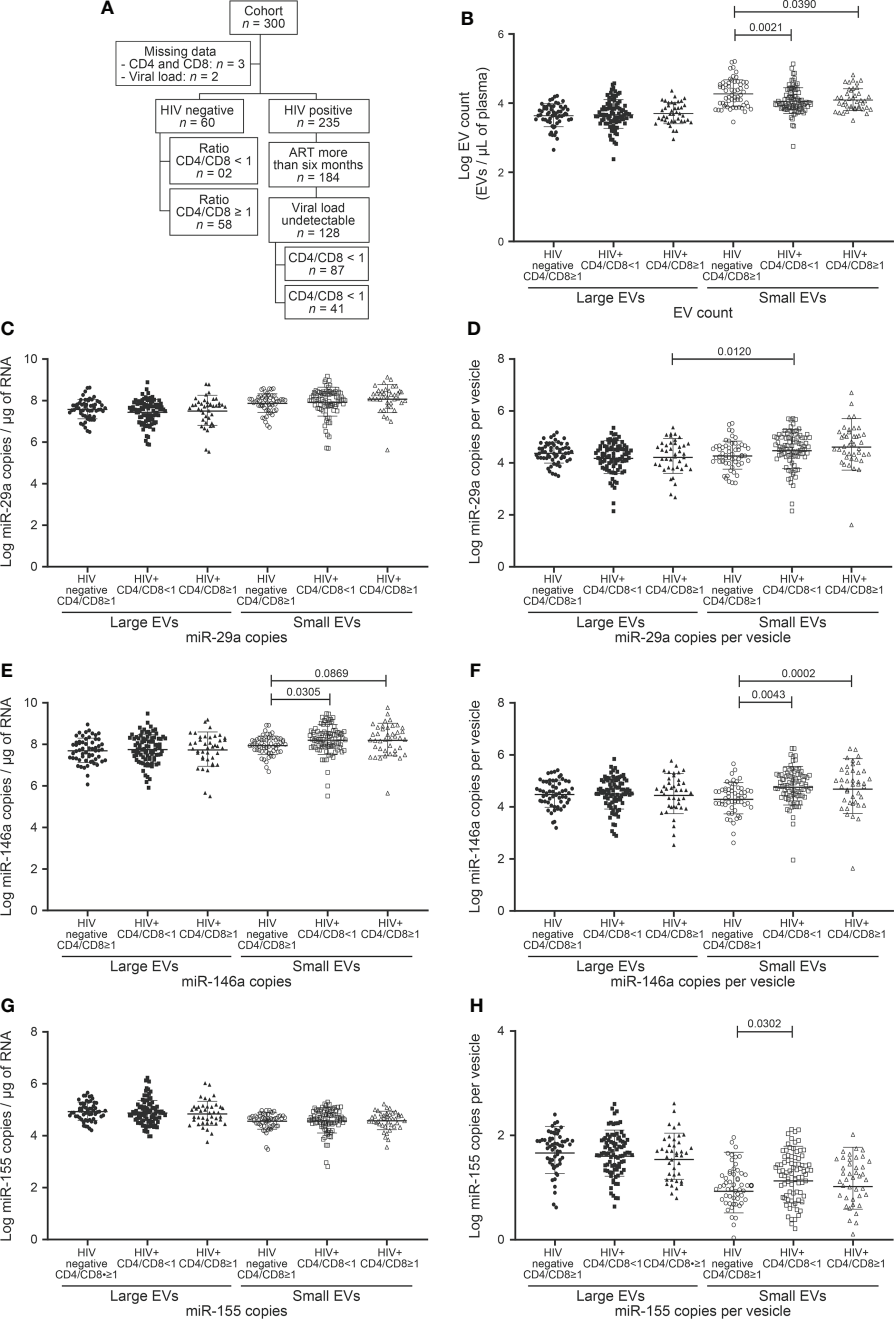
EVs count and miRNAs expression in study participants following to ratio CD4/CD8 T cells count. **(A)** Flow chart of study participants included in the analysis based on ratio CD4/CD8 T cells count. **(B)** Large and small EVs particles number quantification in flow cytometry analysis. **(C, D)**, **(E, F)**, and **(G, H)** present respectively EVs miR-29a, miR-146a, and miR-155 quantification and expression as copies per µg of RNA and copies per vesicle. An ordinary one-way ANOVA with Tukey’s multiple comparisons test was used for comparison between groups.

**Figure 3:**
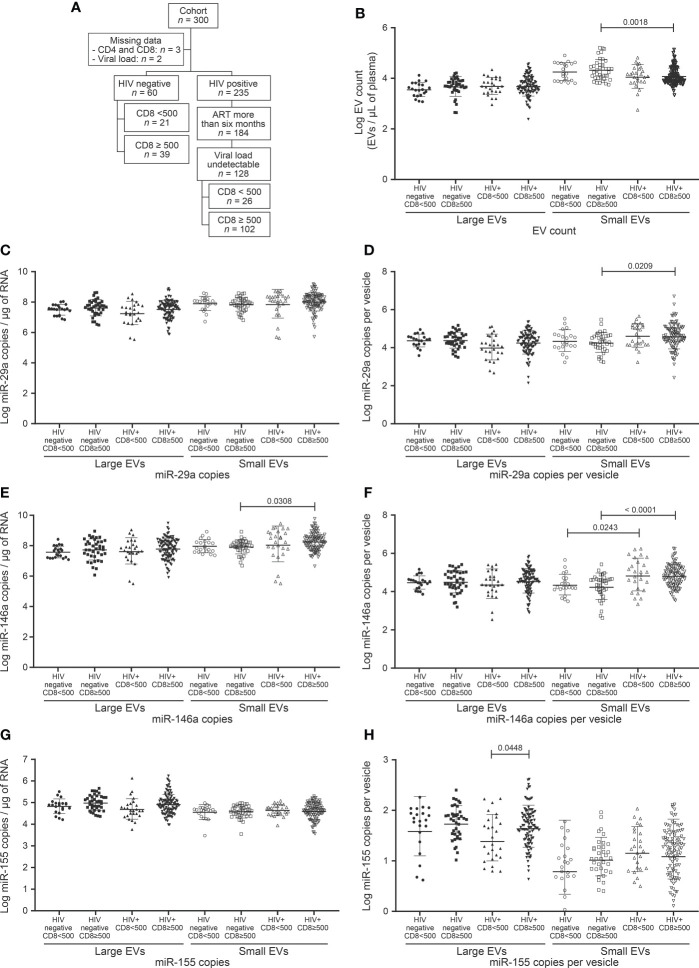
EV count and miRNAs expression in study participants following to CD8 T cells count. **(A)** Flow chart of study participants included in the analysis based on CD8 T cells count. **(B)** Large and small EVs particles number quantification in flow cytometry analysis. **(C, D)**, **(E, F)**, and **(G, H)** present respectively EVs miR-29a, miR-146a, and miR-155 quantification and expression as copies per µg of RNA and copies per vesicle. An ordinary one-way ANOVA with Tukey’s multiple comparisons test was used for comparison between groups.

The CD4/CD8 ratio has previously been found strongly correlated with immune activation and immune senescence (31, 32). It appears to be a good predictor of AIDS and non-AIDS clinical syndromes associated with HIV-1 infection. The discovery of functional biomarkers easier to measure would provide means of improving the care of PLWH. We, therefore, checked large and small EV counts and miRNAs contents for concordance with the CD4 count (**Figure 1**), CD4/CD8 ratio (**Figure 2**), and CD8 count (**Figure 3**) in patients treated with ART for over six months and having undetectable viremia.

Failing to direct measure of cellular and soluble markers of immune activation, a CD4/CD8 ratio < 1 and a CD8 T-cell count > 500/µL were considered as markers of immune activation in this study. Indeed, CD4/CD8 ratio < 1 is a known marker of immune activation and is associated with the activated T cell phenotype characterized by CD38 and HLA-DR expression (31–33). The persistent abnormally high CD8 T cell count in ART-treated PLWH is regarded as a stigma of immune activation (34, 35). Whereas a CD4 count > 500 does indicate immune restoration, CD8 > 500 can give a ratio equal to 1. In the absence of a reference threshold for CD8 count as there is for CD4, we decided to use 500 cells (**Figure 3**).

Demographic and clinical characteristics of the participants are summarized as proportions or medians with interquartile range in **Table 1**. The HIV+ group (n = 128) consisted entirely of persons who had received antiretroviral treatment for more than six months and had undetectable viremia (HIV-1 RNA < 20 copies/mL). Sixty uninfected persons participated as control subjects. The median CD4 T cell counts were respectively 992 (Interquartile range (IQR) 794–1276) and 513 (IQR 386–755) cells/µL (P < 0.0001) for uninfected and HIV infected participants. The median CD8 T cell counts were 584 (IQR 447–715) and 736 (IQR 536–1093) per µL (P = 0.0003). The median CD4/CD8 ratios were 1.8 (IQR 1.4–2.1) and 0.7 (IQR 0.5–1.0) with P < 0.0001. The median length of time since HIV diagnosis was 55 (24–120) months and the median duration on ART was 38 (IQR 21–96) months.

Since a CD4 T cell count below 500 per µL is associated with high morbidity and mortality even in non-viremic patients on ART, we assessed the impact of CD4 T cell count below and above this level on plasma EV counts and the miRNA contents thereof. HIV+ participants were divided into two groups based on CD4 count (**Table 1**, **Figure 1A**). Unexpectedly, neither large nor small EVs were more abundant due to this dichotomy (**Figure 1B**). However, small EVs were significantly more abundant in control participants than HIV+ participants (**Figure 1B**). Only miR-29a was shown to be more abundant in HIV+ patients with CD4 count ≥ 500 cells/µL than in those with lower CD4 counts (**Figures 1C–H**). Compared to control participants, HIV+ patients exhibited stronger expression of miR-146a in small EVs (**Figures 1E, F**). We further divided HIV+ participants with CD4 T cell count < 500 into < 350 and 350–500 (**Supplementary Figure 1**). We observed a great expression of miR-29a (copies per vesicle) in large EVs in the 350–500 category (**Supplementary Figure 1C**). These results show overall that CD4 T cell deficit by itself had no significant impact on EV count or miR-146a and miR-155 contents, and miR-29a is overexpressed in small EVs in HIV+ individuals with higher CD4 counts.

We, therefore, checked large and small EV counts and miRNAs contents for concordance with the CD4/CD8 ratio dichotomized < 1 and ≥ 1 (**Table 1**, **Figure 2A**). As may be surmised in **Figure 2** and **Supplementary Figure 2**, no EV analyses appear to qualify as a proxy for this CD4/CD8 categorization. As observed for CD4 T cell counts, only the miR-146 content of small EVs differed significantly between control subjects and HIV+ patients (**Figures 2E, F**). Copies of miR-155 were more abundant in small EVs of HIV+ patients with a ratio < 1 than those of control participants with a ratio ≥ 1 (**Figure 2H**). These results show a non-significant variation of EV count and miRNA content in HIV+ patients according to CD4/CD8 ratio and hence the need to evaluate other parameters for immune activation assessment.

Chronic HIV infection is characterized by increasing counts of circulating CD8 T cells that persist long after ART. The relationship between CD8 count and EV (abundance and miRNA content), were investigated in participants, which were divided into 2 classes, based on CD8 T cell count < 500/µL and ≥ 500/µL (**Table 1**, **Figure 3A**). This threshold was chosen since 500/µL is the lowest CD4 T cell count considered healthy, a CD4/CD8 ratio of 1. We saw no apparent association between CD8 status and large EV count, miR 29a or miR 146a content (**Figures 3B–F**). However, miR 155 copies per large EV were significantly higher in HIV+ patients with ≥ 500 CD8 T cells/µL (**Figures 3G, H**). Expression of miR-29a and miR-146a heightened in small EVs in this HIV+ group compared to the control participants with ≥ 500 CD8 T cells per µL of blood (**Figures 3D–F**). HIV+ participants with ≥ 500 CD8 T cells/µL were grouped into 500–1,000/µL and ≥ 1,000/µL classes (**Supplementary Figure 3**). We observed that miR-155 was significantly more abundant in large EVs in 500–1,000/µL compared to the < 500/µL group (**Supplementary Figure 3F, G**). These results show an overall increase in miR-155 in large EVs of HIV+ individuals with a CD8 T cell count ≥ 500 cells/µL.

**Figure 4:**
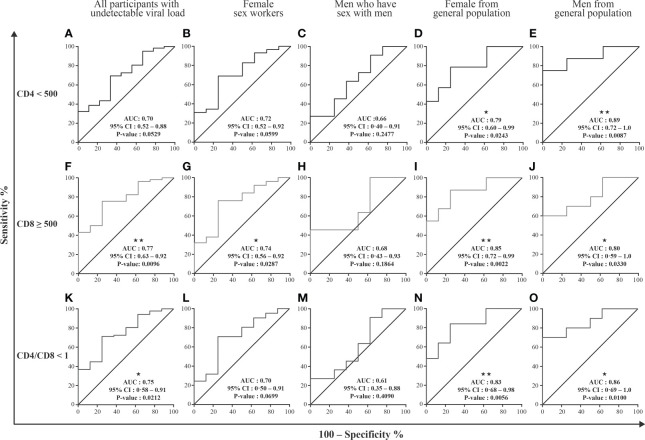
EVs miRNAs content diagnosis performance in receiver operating characteristics curves analysis. Large and small EVs miRNA content was used to generate a Receiver Operator Characteristic (ROC) curve analysis to discriminate participants with different conditions. The participants with CD8 T cell < 500 cells/µL, CD4 T cell ≥ 500 cells/µL, and ratio CD4/CD8 ≥ 1 (group 1, n=10) were used as controls. Diagnosis performance of large EVs miR-155 copies for the discrimination of all participants **(A, F, K)**, female sex workers **(B, G, L)**, men who have sex with men **(C, H, M)**, and female **(D, I, N)** and men **(E, J, O)** from general population with respectively CD4 T cell count ≤ 500 cells/µL, ratio CD4/CD8 < 1, CD8 T cell count ≥ 500 cells/µL. Wilson/Brown method was used to compute the area under a ROC curve.

We next studied the possibility of discriminating between specific HIV-related conditions, namely CD4 T cell count < 500/µL, CD8 T cell count ≥ 500/µL and CD4/CD8 ≤ 1, based on analysis of the miRNA content of EVs. Receiver operator characteristic (ROC) curve analysis was performed using as controls HIV+ individuals ART-treated for over six months and having undetectable viremia with CD8 T cell < 500 cells/µL and CD4 T cell ≥ 500 cells/µL (**Supplementary Tables 1–5**). Only large EVs miR-155 copies distinguished participants with immune activation (**Supplementary Table 1**, **Figures 4A, F, K**), with AUC of 0.75 for CD4/CD8 < 1 (95% CI: 0.58–0.91, P = 0.0212) and 0.77 for CD8 T cells ≥ 500/µL (95% CI: 0.63–0.92, P = 0.0096). In addition, these measured are significant for female sex workers, men and women from the general population (**Figure 4**, **Supplementary Tables 2–5**). These analyses collectively suggest that the miR-155 in large EVs, could constitute the best currently known biomarker for diagnosing immune activation and managing this condition in HIV+ patients.

### EVs miRNA content can cluster ART-treated PLWH with undetectable viral load

**Figure 5:**
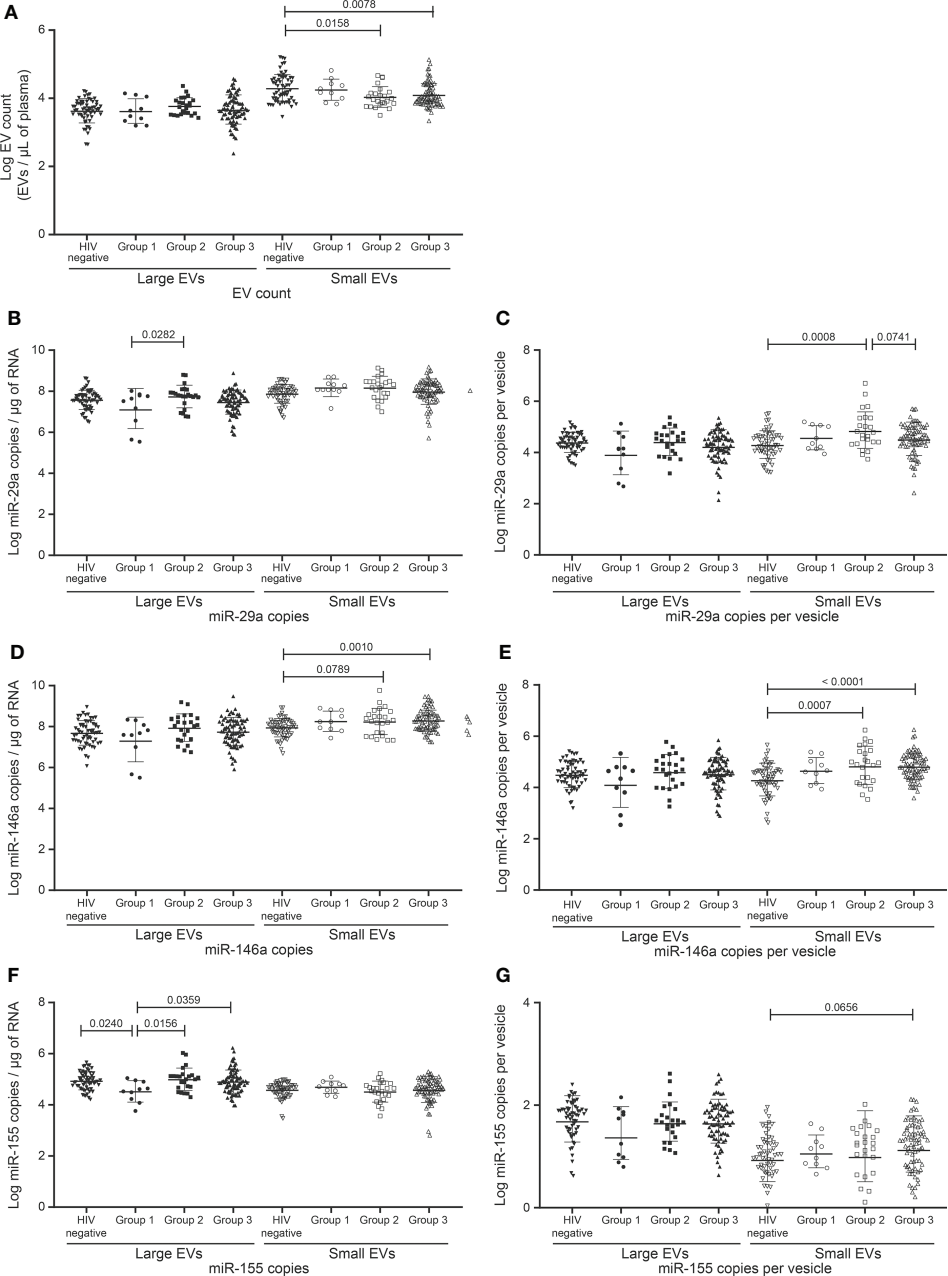
Change in the EVs count and miRNAs expression in study participants Follow a combination of undetectable viral load, CD8 T cell count, and CD4/CD8 ratio. HIV infected participants on ART for more than six months and with undetectable viral load (< 20 copies/mL) were divided into three groups. The reference group is characterized by participants with CD8 T cell < 500 cells/µL, CD4 T cell ≥ 500 cells/µL, and ratio CD4/CD8 ≥ 1 (group 1, n=10). The second group is characterized by ratio CD4/CD8 ≥ 1 and CD8 T cell ≥ 500 cells/µL (group 2, n = 24), and the last group is characterized by ratio CD4/CD8 < 1 and CD8 T cell ≥ 500 cells/µL (group 3, n = 78). **(A**) Large and small EVs particles number quantification in flow cytometry analysis. **(B–E)**, and **(F, G)** present respectively EVs miR-29a, miR-146a, and miR-155 quantification and expression as copies per µg of RNA and copies per vesicle. An ordinary one-way ANOVA with Tukey’s multiple comparisons test was used for comparison between groups.

To further investigate the possibility of using EV analysis in managing immune activation and inflammation in non-viremic persons, the HIV+ participants were divided into three classes based on CD8 T cell count. Those with fewer than 500 CD8 T cells/µL and at least 500 CD4 T cells/µL comprised group 1 (n = 10), those with at least 500 CD8 T cells/µL but CD4/CD8 > 1 comprised group 2 (n = 24), and those with at least 500 CD8 T cells/µL and CD4/CD8 < 1 group 3 (n = 78). EV counts did not differ significantly between these groups but were lower in groups 2 and 3 than in control (not infected) participants (**Figure 5A**).

One microRNA species was found significantly more abundant in groups 2 and 3 compared to group 1, namely miR-155 in large EVs (**Figures 5B–G**). In small EVs, miR-146a tended to be more abundant in groups 2 and 3 than in control participants (**Figures 5D, E**). In the case of miR-155 copies per large vesicle, group 1 appeared to be split into two subpopulations (**Figure 5G**). The bottom subpopulation differed significantly from groups 2 and 3 in miR-155 copies per vesicle (data not shown). Group 1 miR-155 in large EVs also differed from that of the control group. These results overall reinforce our observations of large-EV-associated miR-155 as a possible biomarker of immune activation.

**Figure 6:**
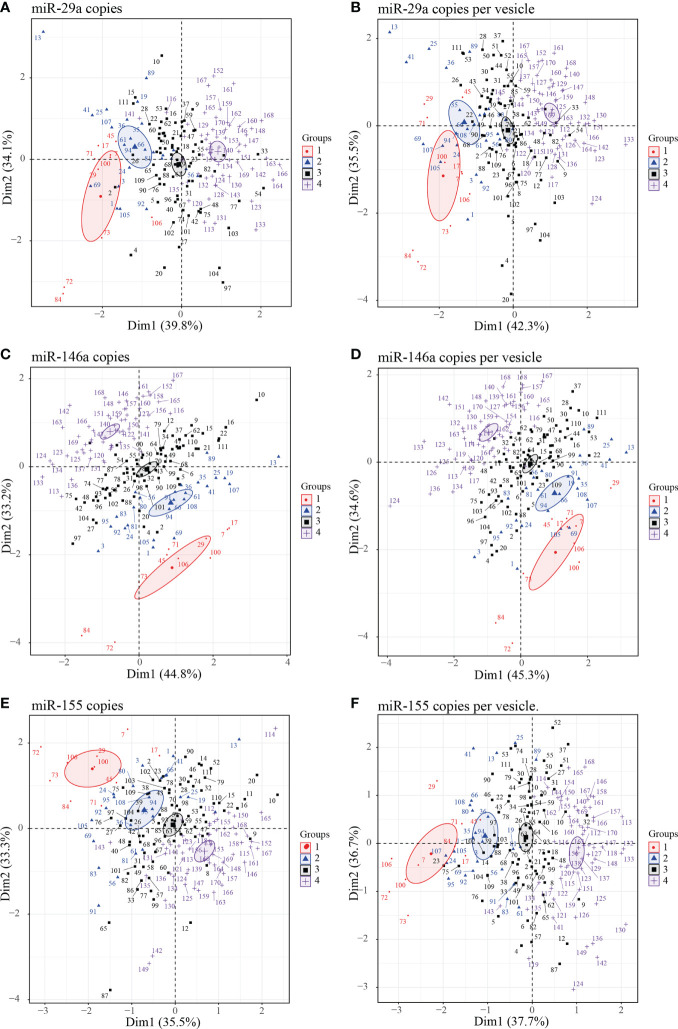
Principal component analysis (PCA) plots of individuals large and small EVs microRNA content. Graphs of individual large and small EVs miR-29a **(A, B)**, miR-146a **(C, D)** and miR-155 **(E, F)** content respectively expressed as copies per µg of RNA and copies per vesicle were performed in PCA for HIV negative and HIV+ ART-treated participants with undetectable viral load participants divided into four groups. Group 1 (participants with CD8 T cell < 500 cells/µL, CD4 T cell ≥ 500 cells/µL, and ratio CD4/CD8 ≥ 1); group 2 (participants with ratio CD4/CD8 ≥ 1 and CD8 T cell ≥ 500 cells/µL), group 3 (participants with CD4/CD8 < 1 and CD8 T cell ≥ 500 cells/µL), and group 4 (HIV negative independently to CD4 or CD8 T cells count and ratio CD4/CD8). Ellipses were drawn around the centroids of the clusters, representing 95% confidence intervals.

To further explore the performances of EVs miRNA content to distinguish different groups of PLWH based on CD4 T cells count, CD8 T cells count, and ratio CD4/CD8, we performed a principal component analysis (PCA) including both HIV infected and uninfected subjects (the latter forming group 4) to evaluate the correlation between individuals. Taking together the expression of the three microRNAs in the large and small EVs there is more clear separation concerning miRNA amount as copies per vesicle between uninfected participants and those infected with HIV (**Supplementary Figure 4**). Focusing on the three miRNAs expression independently in large and small EVs, we observe a better graphical representation of individuals and groups with the large EVs miRNAs (**Supplementary Figure 5**). Finally, regarding the expression of each miRNA together in large and small EVs, miR-146a (**Figures 6C, D**) and miR-155 (**Figures 6E, F**) better cluster individuals from different groups than miR-29a (**Figures 6A, B**). The expression of miR-155 as copies/µg of RNA seemed to better cluster in group 1 individuals than other miRNAs. Collectively, these results comfort the role that the expression of miRNAs in large and small EVs could play in monitoring infected people.

## Discussion

The determinant of immune activation in individuals infected with HIV is well known. Nevertheless, the identification of biomarkers potentially involved in mechanism under this dysregulation is inexistent. Here, we investigated plasma EVs and their microRNA contents, specifically miR-29a, miR-146a, and miR-155, as possible biomarkers of immune activation in PLWH. As observed over two independent cohorts (11, 17), the differential abundance of miRNA species in large and small EVs confirms that EV heterogeneity is not limited to origin but includes functional states. The mechanisms by which different EV subtypes become differentially loaded with specific miRNA molecules are largely unknown and must be elucidated. Meanwhile, evidence is accumulating that miR-155 is more abundant in large EVs of HIV+ individuals and that it is currently the best diagnostic miRNA for recognizing patients experiencing immune activation based on CD8 T cell count and CD4/CD8 ratio.

In its global strategy to end the AIDS epidemic by 2030, UNAIDS aims to treat 95% of people aware of their infection and suppress viremia in 95% of people treated. Since antiviral drugs do not target immune activation and residual immune activation persists despite suppression of viremia, determining the level of chronic immune activation and reducing its negative impact have become essential aspects of optimizing ART. Activation of the cell-mediated immune response has been associated with changes in the expression of specific microRNA molecules. In view of previously noted correlations between counts of CD4 and CD8 T lymphocytes and extracellular vesicle size distribution (11, 17), it made sense to examine the amount of miR-29a, miR-146a, and miR-155 in large and small vesicles under different HIV-related conditions.

Previous studies of peripheral blood mononuclear cells (PBMCs) have suggested that miR-29a level is inversely correlated with viremia, that its overexpression is associated with inhibition of viral replication and could promote viral latency (13, 14). Earlier studies of cellular immune dysfunction in persons infected with HIV have shown increased miR-146a and miR-155 expression in PBMCs. Again, we observed no variation in EV-borne miR-29a and miR-146a levels associated with HIV-related changes in CD4 and CD8 T cell counts or CD4/CD8 ratio. Expression of miR-146a in small EVs was significantly stronger in non-viremic HIV+ individuals than in control subjects, which has been attributed to inflammation and oxidative stress (36). Elevated expression of miR-146a reportedly correlates with high levels of immune cell exhaustion markers and suppresses cellular immune function in chronic HIV-1-infected patients (15). HIV infection is characterized by persistent high percentages of effector and effector memory cells, possibly explaining miR-146a overexpression in small EVs, and suggesting this molecule as a marker of inflammation.

In this study, the best proxy candidate for CD4 and CD8 T cell counts as clinical parameters for monitoring HIV infection appears to be miR-155 in large EVs. High levels of miR-155 have been found previously in total PBMCs, CD4 T cells and CD8 T cells in HIV-1-infected patients, and its expression on T cells has even been correlated with immune activation (16). CD8+ T cells activated *in vitro* with solid-phase anti-CD3/anti-CD28 antibodies for 24 h to 5 days can increase miR-155 expression by 42 to 104 fold compared to unstimulated naive CD8+ T cells (37). Since activated cells are known to secrete vesicles containing increased amounts of microRNA, we hypothesized that CD8 T lymphocytes could produce large amounts of miR-155-rich EVs during HIV infection. It has been found that the persistent antigenic stimulation that characterizes HIV-1 infection contributes to immune activation and induces increased expression of miR-155 in correlation with disease severity (38). MiR-155 overexpression associated with chronic viral infection leads to increased persistence of CD8 T cells and correlates with higher expression of inhibitory receptors on these cells (39). MiR-155-loaded EVs may contribute to the maintenance of immune activation and inflammation observed in PLWH despite ART, because of the pleotropic action of miR-155 on other immune cells (40), thus maintaining the immune activation loop.

Based on ROC curve analysis, miR-155 expression in large EVs was the best predictor of immune activation marked by elevated CD8 T cell count and low CD4/CD8 ratio. Looking at our participants’ subgroups, namely female sex workers, men who have sex with men, women and men from the general population, these results are verified in 3/4 of cases. The lack of significance in the group of men who have sex with men could be explained by their relatively small number in this analysis, thus leading to lack of statistical power to detect associations in this subgroup. These results support the validity of this new biomarker for discriminating HIV-infected patients with a certain degree of immune activation.

One might also wonder if this overexpression of miR-155 is not related to inflammation rather immune activation. The miR-155 and miR-146a species have been associated with a variety of pathological conditions characterized by chronic inflammation (41, 42). Despite their involvement in the inflammatory process at different levels, epistasis between them has been confirmed, and miR-155 reportedly dominates in both CD4+ and CD8+ T cell-mediated antitumor immunity (43). Obviously, the lack of significant results with miR-146a is in favor of immune activation.

## Conclusions

Since the molecular composition of EVs closely reflects that of their secreting cells, they should indicate a functional state of immune cells. Their use as liquid biopsy should allow researchers to elucidate mechanisms of HIV infection pathophysiology and help clinicians monitor patient status. Their microRNA load tells us not only which cells likely produced them but also for what biological process, including immune activation and inflammation. Combinations of plasma and cellular activation biomarkers should be studied to confirm and improve their use as diagnostic parameters. Large EVs with miR-155 need to be examined more closely as a reliable, functional biomarker of immune activation.

## Data availability statement

De-identified participant data from this study and corresponding data dictionary, study protocol, and informed consent documents will be made available to researchers upon request to the corresponding author. Researchers will be asked to complete a concept sheet for their proposed analyses to be reviewed, and the investigators will consider the overlap of the proposed project with active or planned analyses and the appropriateness of study data for the proposed analysis.

## Ethics statement

The studies involving human participants were reviewed and approved by Burkina Faso Health Research Ethics Committee (N 2017-12-182, December 12, 2017) and Centre de Recherche du CHU de Québec-Université Laval (Québec, Canada) research ethics board (Project 2012-890, C12-03-167/Modification F1 – 32940 2018-08-31). The patients/participants provided their written informed consent to participate in this study.

## Author contributions

Conceived and designed the experiments: WW and CG; Performed the experiments: WW, CG, JB, and BG; Analyzed the data WW and CG; Contributed clinical samples, reagents, materials, and analytical tools: WW, IT, DK, DS, MA, and CG; Wrote the manuscript: WW and CG. All authors have critically reviewed the paper and have agreed on the published version of the manuscript.

## Funding

The funding sources had no hand in the study design. This research was funded through Canadian Institutes of Health Research (CIHR) grants MOP-188726; MOP-267056 (HIV/AIDS initiative) to CG and the Canadian Institutes of Health Research CIHR Foundation Grant FDN-143218 to MA for the studentship awarded to WW. WW is the recipient of the leadership and sustainable development scholarship and the Fonds de recherche du Québec – Santé (FRQ-S) doctoral training scholarship. WW and JB are recipients of the recruitment scholarship from the AIDS Research Fund of Université Laval, and the Desjardins scholarship from the Fondation du CHU de Québec. The FRQ-S supports the Centre de recherche du CHU de Québec – Université Laval infrastructure.

## Acknowledgments

The authors thank Dr. Stephen Davids for his assistance in editing this manuscript, Drs. Martin Pelletier and Stephane Gobeil for access to the qPCR platform. We acknowledge the Bioimaging platform of the Infectious Disease Research Center, and cytometry platform funded by an equipment and infrastructure grant from the Canadian Foundation for Innovation (CFI) and Julie-Christine Levesque for microscopy analysis. We gratefully acknowledge the continuing collab-oration of persons living with HIV. We are particularly grateful to people of patient monitoring centers who allowed and facilitated recruitment, sample collection and analysis: Prof. Joseph Drabo and Martin Bazongo from CHU Yalgado Ouédraogo day hospital, Dr. Elias Dah from the AAS Center, Prof. Armel Poda and Prof. Abdoul Salam Ouédraogo, Leonel Haltolna from CHU Souro Sanou day hospital, Dr. Yacouba Sourabié and Dr. Abdoulaye Semde from the immunology service of the CHU-Souro Sanou, Ousseni Bandaogo and Viviane Nikiema from the virology laboratory of the Centre Muraz, Yago Aziz, Dr. Yvette Zoundi and Karambiri Zakaria from Yerelon clinics. Thanks for your help.

## Conflict of interest

The authors declare that the research was conducted in the absence of any commercial or financial relationships that could be construed as a potential conflict of interest.

## Publisher’s note

All claims expressed in this article are solely those of the authors and do not necessarily represent those of their affiliated organizations, or those of the publisher, the editors and the reviewers. Any product that may be evaluated in this article, or claim that may be made by its manufacturer, is not guaranteed or endorsed by the publisher.
