# Supplementary material for: Plasma vesicular miR-155 as a biomarker of immune activation in antiretroviral treated people living with HIV
Source: Front Immunol. 2022 Aug 29;13:916599. doi: 10.3389/fimmu.2022.916599 (PMC9464867; doi:10.3389/fimmu.2022.916599)
Supplement: Supplementary file 1 [file DataSheet_1.pdf]

frontiers

Supplementary Material

1 Supplementary Data

Supplementary Material should be uploaded separately on submission. Please include any supplementary data, figures and/or tables. All supplementary files are deposited to FigShare for permanent storage and receive a DOI.

Supplementary material is not typeset so please ensure that all information is clearly presented, the appropriate caption is included in the file and not in the manuscript, and that the style conforms to the rest of the article. To avoid discrepancies between the published article and the supplementary material, please do not add the title, author list, affiliations or correspondence in the supplementary files.

2 Supplementary Figures and Tables

For more information on Supplementary Material and for details on the different file types accepted, please see here. Figures, tables, and images will be published under a Creative Commons CC-BY licence and permission must be obtained for use of copyrighted material from other sources (including re-published/adapted/modified/partial figures and images from the internet). It is the responsibility of the authors to acquire the licenses, to follow any citation instructions requested by third-party rights holders, and cover any supplementary charges.

2.1 Supplementary Figures

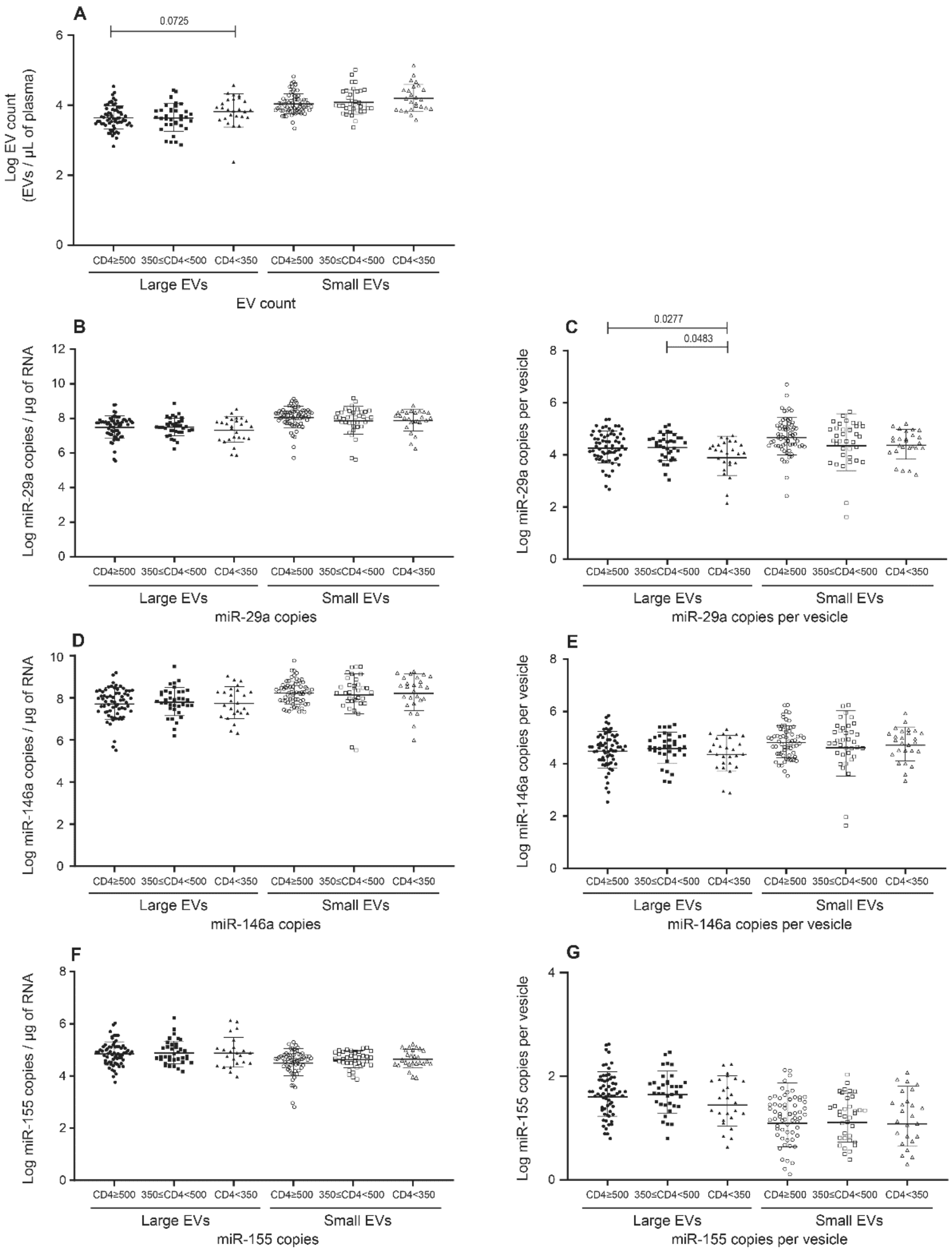

Supplementary Figure 1. Counts of large and small EVs and miRNA contents thereof versus CD4 T cell count per mL of blood in persons living with HIV (non-viremic). (**A**) EV quantification by flow cytometry; **(B through G**) MicroRNA content expressed as copies per µg of total RNA and copies per vesicle. Significant differences between groups are based on ordinary one-way ANOVA with Tukey's multiple comparisons test.

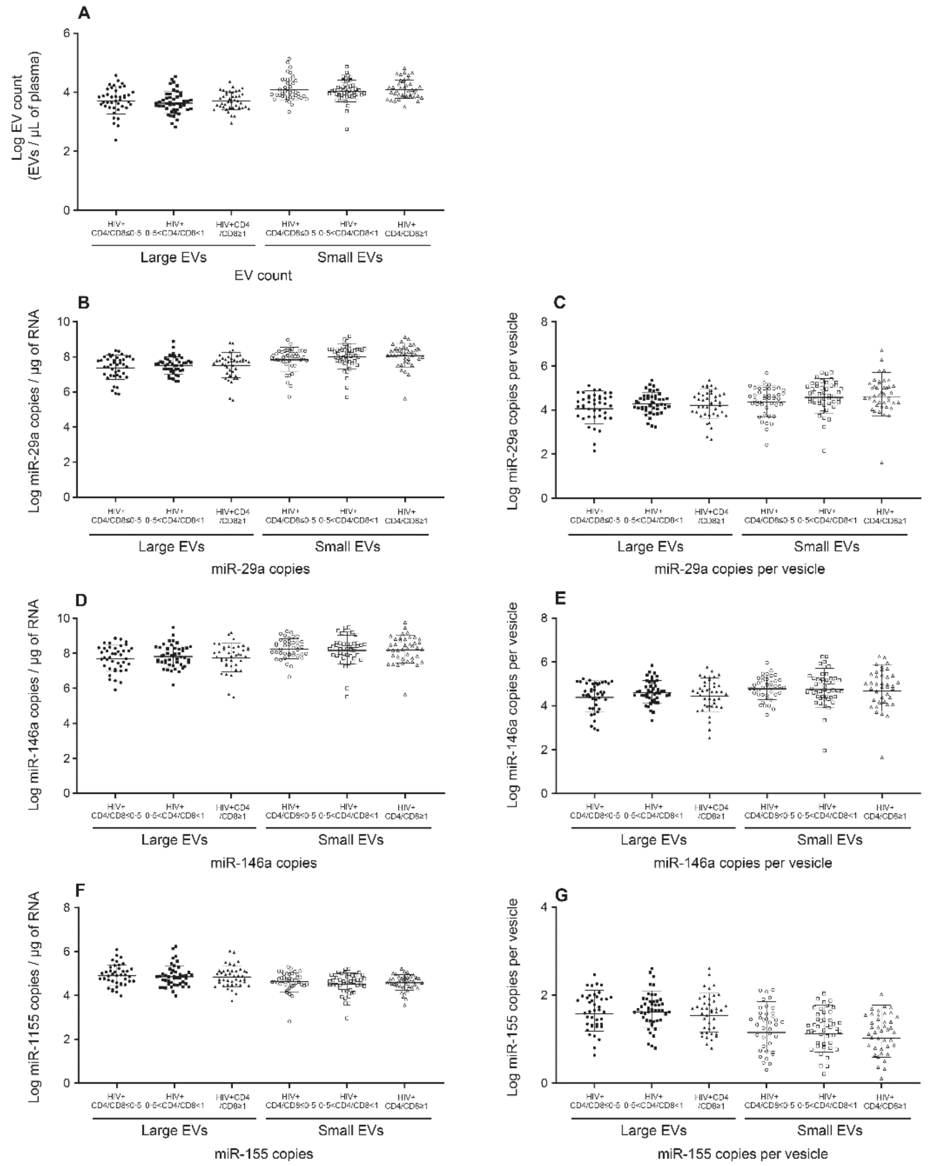

Supplementary Figure 2. Counts of large and small EVs and miRNA contents thereof versus CD4/CD8 T cell ratio in blood of persons living with HIV (non-viremic). **(A**) EV quantification by flow cytometry; **(B through G**) MicroRNA content expressed as copies per µg of total RNA and copies per vesicle. Significant differences between groups are based on ordinary one-way ANOVA with Tukey's multiple comparisons test.

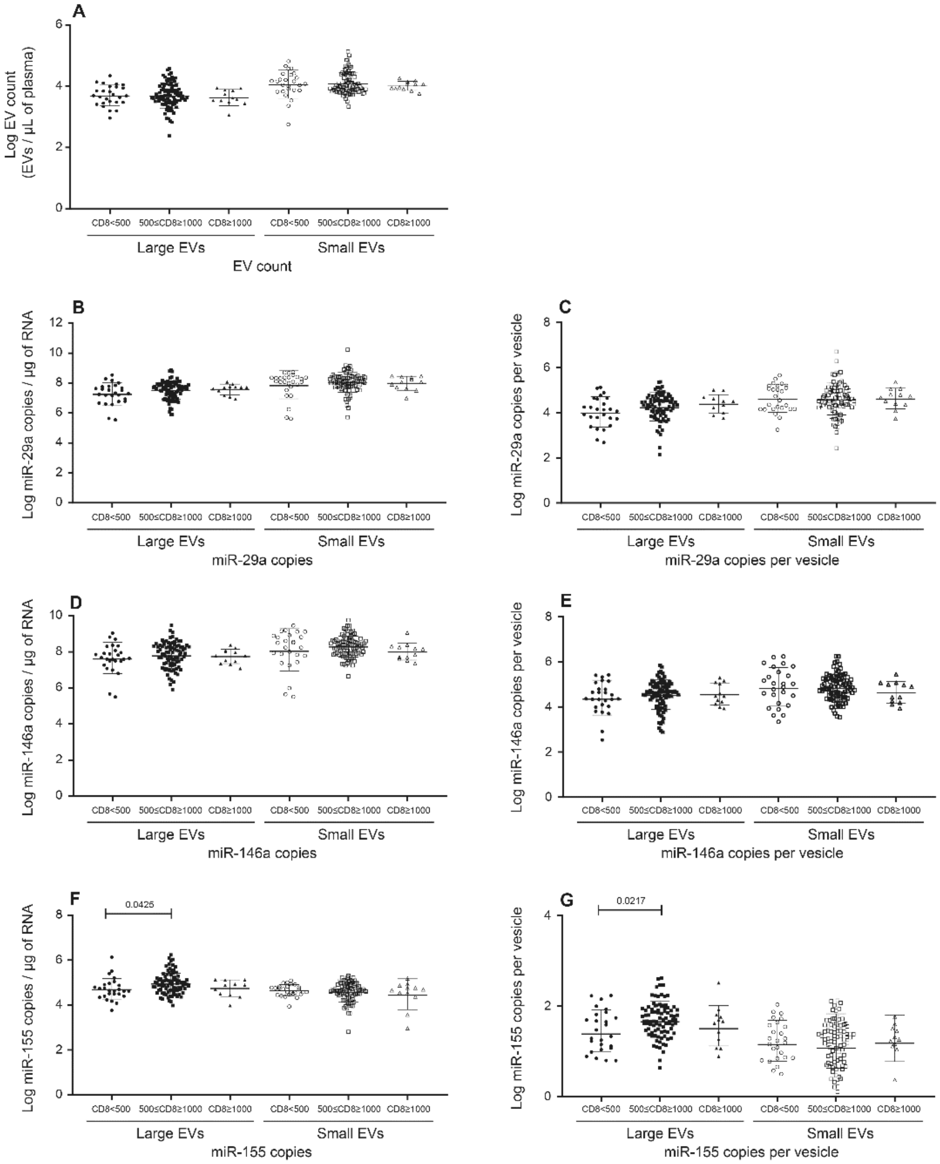

Supplementary Figure 3. Counts of large and small EVs and miRNA contents thereof versus CD8 T cell count ranges in blood of persons living with HIV (non-viremic). **(A)** EV quantification by flow cytometry; (**B through G)** MicroRNA content expressed as copies per µg of total RNA and copies per vesicle. Significant differences between groups are based on ordinary one-way ANOVA with Tukey's multiple comparisons test.

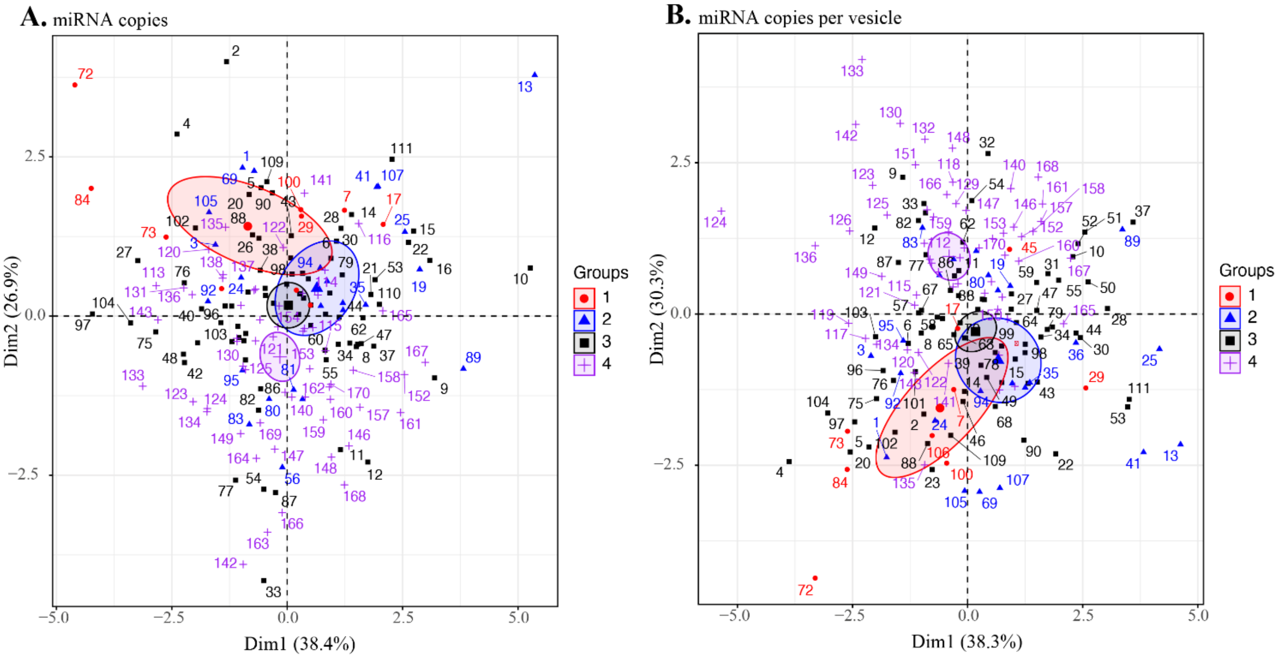

Supplementary Figure 4. Principal component analysis (PCA) plots of individuals EVs miRNA content. Graphs of individual large and small EVs miR-29a, miR-146a and miR-155 content expressed as copies/ µg of RNA (**A**) and copies per vesicle (**B**) were performed in PCA for HIV negative and HIV+ ART-treated participants with undetectable viral load participants divided into four groups. Group 1 (participants with CD8 T cell < 500 cells/µL, CD4 T cell ≥ 500 cells/µL, and ratio CD4/CD8 ≥ 1); group 2 (participants with ratio CD4/CD8 ≥ 1 and CD8 T cell ≥ 500 cells/µL), group 3 (participants with CD4/CD8 < 1 and CD8 T cell ≥ 500 cells/µL), and group 4 (HIV negative control independently to CD4 or CD8 T cells count and ratio CD4/CD8). Ellipses were drawn around the centroids of the clusters, representing 95% confidence intervals.

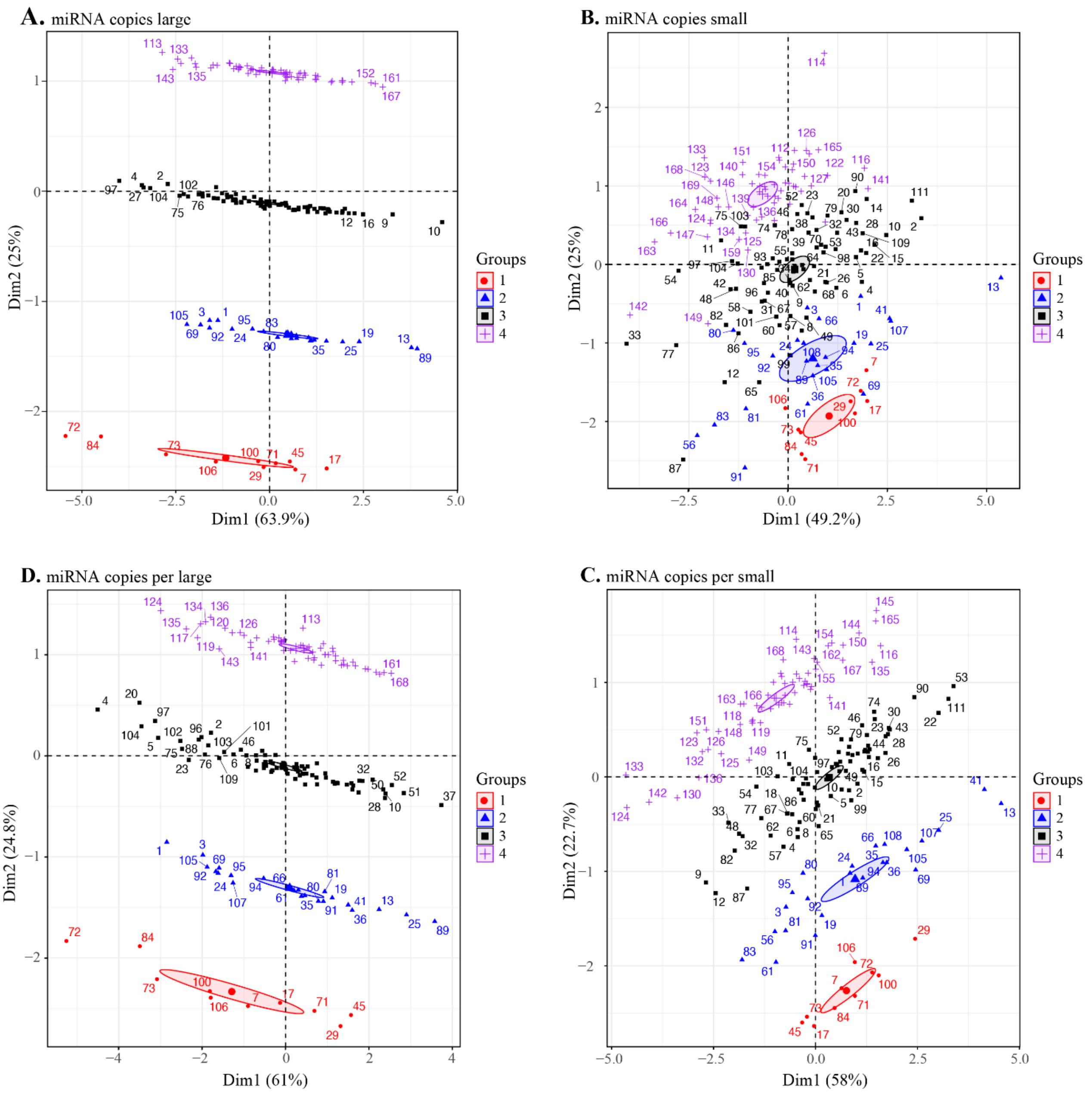

Supplementary Figure 5. Principal component analysis plots of individuals large and small EVs microRNA content. Graphs of individual large and small EVs miR-29a, miR-146a and miR-155 content expressed as copies/µg of RNA (**A and B**), and copies per vesicle (**C and D**) were performed in PCA for HIV negative and HIV+ ART-treated participants with undetectable viral load participants divided into four groups. Group 1 (participants with CD8 T cell $<$ 500 cells/µL, CD4 T cell $\geq$ 500 cells/µL, and ratio CD4/CD8 $\geq$ 1); group 2 (participants with ratio CD4/CD8 $\geq$ 1 and CD8 T cell $\geq$ 500 cells/µL), group 3 (participants with CD4/CD8 $<$ 1 and CD8 T cell $\geq$ 500 cells/µL), and group 4 (HIV uninfected control independently to CD4 or CD8 T cells count and ratio CD4/CD8). Ellipses were drawn around the centroids of the clusters, representing 95% confidence intervals.

3 Supplementary Tables

Supplementary Table 1. Receiver operating characteristics curve analysis of EV miRNA content as a diagnostic of immune activation in HIV+ patients.

	**Patients with CD4 count ≤ 500 cells/µL**		**Patients with CD8 count ≥ 500 cells/µL**		**Patients with CD4/CD8 ratio ≤ 1**	
	**Area under the curve (95% CI)**	**P value**	**Area under the curve (95% CI)**	**P value**	**Area under the curve (95% CI)**	**P value**
miR-29a, large EV, per µg RNA	0.57 (0.36–0.77)	0.5044	0.60 (0.41–0.79)	0.2840	0.57 (0.37–0.77)	0.4840
miR-29a, small EV, per µg RNA	0.61 (0.46–0.77)	0.2351	0.62 (0.50–0.75)	0.1909	0.60 (0.45–0.75)	0.2979
miR-29a copies per large EV	0.57 (0.35–0.78)	0.5044	0.61 (0.40–0.81)	0.2574	0.58 (0.37–0.79)	0.3865
miR-29a copies per small EV	0.52 (0.34–0.71)	0.7945	0.53 (0.34–0.72)	0.7673	0.51 (0.32–0.70)	0.8868
miR-146a, large EV, per µg RNA	0.61 (0.43–0.80)	0.2476	0.62 (0.44–0.79)	0.2247	0.60 (0.41–0.78)	0.3076
miR-146a, small EV, per µg RNA	0.52 (0.36–0.70)	0.7695	0.50 (0.32–0.68)	0.9836	0.51 (0.33–0.69)	0.9236
miR-146a copies per large EV	0.60 (0.40–0.80)	0.2974	0.61 (0.42–0.80)	0.2406	0.60 (0.40–0.80)	0.2965
miR-146a copies per small EV	0.56 (0.39–0.73)	0.5254	0.58 (0.40–0.76)	0.3869	0.58 (0.40–0.76)	0.4079
miR-155, large EV, per µg RNA	0.70 (0.52–0.88)	0.0529	0.77 (0.63–0.92)	0.0096	0.75 (0.58–0.91)	0.0212
miR-155, small EV, per µg RNA	0.52 (0.35–0.69)	0.8324	0.55 (0.39–0.71)	0.6032	0.55 (0.39–0.75)	0.6351
miR-155 copies per large EV	0.59 (0.38–0.81)	0.3450	0.62 (0.40–0.84)	0.2022	0.61 (0.40–0.82)	0.2597
miR-155 copies per small EV	0.57 (0.41–0.73)	0.4670	0.59 (0.43–0.74)	0.3620	0.60 (0.45–0.76)	0.2863

CI: confidence interval; EV: extracellular vesicle

Supplementary Table 2. Receiver operating characteristics curve analysis of EV miRNA content as a diagnostic of immune activation in HIV+ patients for female sex workers subgroup.

	Patients with CD4 count < 500 cells/µL		Patients with CD8 count ≥ 500 cells/µL		Patients with ratio CD4/CD8 count ≤ 1	
	**Area under the curve (95% CI)**	**P value**	**Area under the curve (95% CI)**	**P value**	**Area under the curve (95% CI)**	**P value**
miR-29a, large EV, per µg RNA	0.51 (0.27–0.74)	0.9487	0.53 (0.31–0.75)	0.7661	0.51 (0.28–0.74)	0.9056
miR-29a, small EV, per µg RNA	0.62 (0.44–0.80)	0.2435	0.60 (0.44–0.75)	0.3311	0.56 (0.40–0.74)	0.5086
miR-29a copies per large EV	0.56 (0.33–0.78)	0.6068	0.60 (0.39–0.81)	0.3410	0.57 (0.34–0.79)	0.5218
miR-29a copies per small EV	0.57 (0.35–0.78)	0.5201	0.53 (0.32–0.74)	0.7661	0.52 (0.31–0.74)	0.8125
miR-146a, large EV, per µg RNA	0.52 (0.29–0.74)	0.8722	0.52 (0.31–0.74)	0.8196	0.51 (0.29–0.7)	0.9338
miR-146a, small EV, per µg RNA	0.57 (0.38–0.77)	0.4994	0.56 (0.37–0.76)	0.5256	0.55 (0.35–0.75)	0.6017
miR-146a copies per large EV	0.57 (0.35–0.79)	0.5201	0.56 (0.35–0.77)	0.5518	0.56 (0.34–0.77)	0.5853
miR-146a copies per small EV	0.53 (0.33–0.74)	0.7477	0.52 (0.32–0.73)	0.8273	0.53 (0.32–0.74)	0.7398
miR-155, large EV, per µg RNA	0.72 (0.52–0.92)	0.0599	0.74 (0.56–0.93)	0.0287	0.70 (0.50–0.91)	0.0699
miR-155, small EV, per µg RNA	0.58 (0.38–0.78)	0.4402	0.60 (0.42–0.77)	0.3410	0.57 (0.38–0.76)	0.4914
miR-155 copies per large EV	0.57 (0.33–0.81)	0.5201	0.60 (0.36–0.83)	0.3410	0.57 (0.34–0.81)	0.4766
miR-155 copies per small EV	0.54 (0.34–0.70)	0.8219	0.51 (0.34–0.68)	0.9053	0.53 (0.36–0.70)	0.7398

CI: confidence interval; EV: extracellular vesicle

Supplementary Table 3. Receiver operating characteristics curve analysis of EV miRNA content as a diagnostic of immune activation in HIV+ patients for men who have sex with subgroup.

	**Patients with CD4 count ≤ 500 cells/µL**		**Patients with CD8 count ≥ 500 cells/µL**		**Patients with ratio CD4/CD8 count ≤ 1**	
	**Area under the curve (95% CI)**	**P value**	**Area under the curve (95% CI)**	**P value**	**Area under the curve (95% CI)**	**P value**
miR-29a, large EV, per µg RNA	0.51 (0.24–0.77)	0.9439	0.52 (0.27–0.79)	0.8327	0.54 (0.27–0.80)	0.7782
miR-29a, small EV, per µg RNA	0.50 (0.25–0.76)	0.9738	0.61 (0.35–0.88)	0.3787	0.51 (0.25–0.78)	0.9159
miR-29a copies per large EV	0.55 (0.29–0.82)	0.6727	0.54 (0.27–0.80)	0.7782	0.55 (0.29–0.82)	0.6727
miR-29a copies per small EV	0.65 (0.41–0.90)	0.2313	0.59 (0.34–0.84)	0.4813	0.62 (0.37–0.87)	0.3600
miR-146a, large EV, per µg RNA	0.59 (0.34–0.84)	0.4813	0.55 (0.29–0.81)	0.7248	0.51 (0.24–0.78)	0.9439
miR-146a, small EV, per µg RNA	0.79 (0.59–0.99)	0.0.0242	0.68 (0.45–0.92)	0.1590	0.75 (0.54–0.96)	0.0486
miR-146a copies per large EV	0.51 (0.25–0.77)	0.9439	0.51 (0.25–0.77)	0.9439	0.51 (0.23–0.77)	0.9439
miR-146a copies per small EV	0.85 (0.67–1.0)	0.0075	0.81 (0.62–1.0)	0.0167	0.81 (0.62–1.00)	0.0167
miR-155, large EV, per µg RNA	0.66 (0.40–0.91)	0.2477	0.68 (0.43–0.93)	0.1864	0.61 (0.35–0.88)	0.4090
miR-155, small EV, per µg RNA	0.53 (0.27–0.78)	0.8327	0.57 (0.32–0.83)	0.5732	0.58 (0.32–0.84)	0.5262
miR-155 copies per large EV	0.51 (0.24–0.78)	0.9439	0.53 (0.25–0.80)	0.8327	0.52 (0.25–0.79)	0.8880
miR-155 copies per small EV	0.70 (0.47–0.94)	0.1053	0.72 (0.48–0.96)	0.0910	0.71 (0.47–0.94)	0.1053

CI: confidence interval; EV: extracellular vesicle

Supplementary Table 4. Receiver operating characteristics curve analysis of EV miRNA content as a diagnostic of immune activation in HIV+ patients for female from general population subgroup

	Patients with CD4 count ≤ 500 cells/µL		Patients with CD8 count ≥ 500 cells/µL		Patients with ratio CD4/CD8 count ≤ 1	
	**Area under the curve (95% CI)**	**P value**	**Area under the curve (95% CI)**	**P value**	**Area under the curve (95% CI)**	**P value**
miR-29a, large EV, per µg RNA	0.73 (0.53–0.93)	0.06104	0.75 (0.59–0.92)	0.0165	0.72 (0.53–0.90)	0.0486
miR-29a, small EV, per µg RNA	0.62 (0.39–0.85)	0.3195	0.61 (0.43–0.78)	0.3091	0.62 (0.43–0.81)	0.2571
miR-29a copies per large EV	0.73 (0.50–0.96)	0.0610	0.74 (0.53–0.95)	0.0228	0.72 (0.51–0.94)	0.0409
miR-29a copies per small EV	0.54 (0.30–0.78)	0.7253	0.54 (0.34–0.74)	0.7156	0.50 (0.29–0.72)	0.9709
miR-146a, large EV, per µg RNA	0.77 (0.58–0.96)	0.0261	0.79 (0.63–0.94)	0.0069	0.74 (0.56–0.93)	0.0259
miR-146a, small EV, per µg RNA	0.50 (0.26–0.74)	0.9999	0.52 (0.32–0.73)	0.8269	0.50 (0.29–0.71)	0.9999
miR-146a copies per large EV	0.74 (0.56–0.96)	0.0303	0.78 (0.59–0.96)	0.0090	0.74 (0.54–0.95)	0.0259
miR-146a copies per small EV	0.51 (0.27–0.75)	0.9068	0.61 (0.42–0.80)	0.3175	0.57 (0.37–0.77)	0.5205
miR-155, large EV, per µg RNA	0.79 (0.60–0.99)	0.0243	0.85 (0.72–0.99)	0.0022	0.83 (0.68–0.98)	0.0056
miR-155, small EV, per µg RNA	0.56 (0.32–0.79)	0.6395	0.55 (0.36–0.74)	0.6164	0.55 (0.35–0.75)	0.6613
miR-155 copies per large EV	0.71 (0.49–0.93)	0.0895	0.73 (0.53–0.92)	0.0336	0.73 (0.53–0.92)	0.0374
miR-155 copies per small EV	0.67 (0.45–0.91)	0.1538	0.62 (0.43–0.80)	0.2744	0.62 (0.43–0.82)	0.2577

CI: confidence interval; EV: extracellular vesicle

Supplementary Table 5. Receiver operating characteristics curve analysis of EV miRNA content as a diagnostic of immune activation in HIV+ patients for men from general population subgroup

	Patients with CD4 count ≤ 500 cells/µL		Patients with CD8 count ≥ 500 cells/µL		Patients with ratio CD4/CD8 count ≤ 1	
	**Area under the curve (95% CI)**	**P value**	**Area under the curve (95% CI)**	**P value**	**Area under the curve (95% CI)**	**P value**
miR-29a, large EV, per µg RNA	0.65 (0.39–0.91)	0.2863	0.58 (0.32–0.84)	0.5453	0.64 (0.39–0.89)	0.2899
miR-29a, small EV, per µg RNA	0.73 (0.49–0.96)	0.0986	0.78 (0.57–0.99) *	0.0412	0.83 (0.65–1.0) *	0.0143
miR-29a copies per large EV	0.51 (0.23–0.79)	0.9292	0.58 (0.32–0.84)	0.5453	0.54 (0.28–0.80)	0.7624
miR-29a copies per small EV	0.59 (0.31–0.86)	0.5340	0.58 (0.31–0.85)	0.5453	0.62 (0.36–0.88)	0.3643
miR-146a, large EV, per µg RNA	0.73 (0.47–0.98)	0.1097	0.63 (0.37–0.89)	0.3258	0.70 (0.46–0.94)	0.1306
miR-146a, small EV, per µg RNA	0.59 (0.30–0.87)	0.5340	0.53 (0.27–0.74)	0.8206	0.52 (0.26–0.78)	0.8798
miR-146a copies per large EV	0.58 (0.30–0.84)	0.5940	0.50 (0.23–0.77)	0.9999	0.55 (0.29–0.81)	0.7055
miR-146a copies per small EV	0.61 (0.34–0.89)	0.4239	0.57 (0.31–0.83)	0.5963	0.54 (0.28–0.80)	0.7624
miR-155, large EV, per µg RNA	0.89 (0.72–1.0)	0.0087	0.80 (0.59–1.0)	0.0330	0.86 (0.69–1.0)	0.0100
miR-155, small EV, per µg RNA	0.50 (0.19–0.81)	0.9999	0.54 (0.28–0.81)	0.7624	0.58 (0.30–0.86)	0.5453
miR-155 copies per large EV	0.62 (0.35–0.90)	0.3743	0.54 (0.27–0.81)	0.7624	0.60 (0.34–0.86)	0.4497
miR-155 copies per small EV	0.56 (0.26–0.87)	0.6569	0.55 (0.28–0.83)	0.6831	0.52 (0.24–0.80)	0.8798

CI: confidence interval; EV: extracellular vesicle; * Values are higher in the control
